# Effect of Uniaxial Compressive Stress on Phase Transformation Kinetics of Low-Carbon Steel

**DOI:** 10.3390/ma15134477

**Published:** 2022-06-25

**Authors:** Shanchao Zuo, Peng Cheng, Decheng Wang, Bing Du, Keming Guan, Jing Zhang

**Affiliations:** 1 China Productivity Center for Machinery, China Academy of Machinery Science and Technology, Beijing 100048, China; b20170183@xs.ustb.edu.cn (S.Z.); wangdc@cam.com.cn (D.W.); dub@cam.com.cn (B.D.); mingkeguan@sina.com (K.G.); 13811074601@126.com (J.Z.); 2 School of Materials Science and Engineering, University of Science and Technology Beijing, Beijing 100083, China

**Keywords:** uniaxial compressive stress, dilatometric curves, lever principle, the Johnson–Mehl–Avrami (JMA) model

## Abstract

To attain microstructure transformation and the kinetics of phase transformation under stress during the annealing process, dilatometric curves of phase transformation for Q235 steel were tested using a Gleeble-3500 thermal-mechanical simulator under different uniaxial compressive stresses. The Johnson–Mehl–Avrami (JMA) model considering impingement correction was applied to study the phase-transformation kinetics during annealing. The results showed that the grain size increased with increasing uniaxial compressive stresses because it provided additional energy for grain growth. Furthermore, the interfacial migration velocity decreased with increasing stress owing to grain coarsening and a decrease in the density of the α/γ boundary. Meanwhile, the stress reduces the sum of the misfit accommodation energy and interface energy caused by the transformation, and the driving force required for the transformation of austenite to ferrite decreases. Hence, it was concluded that uniaxial compressive stress plays a complex role in the phase transformation, which inhibits interfacial migration and the transformation rate while providing additional energy for the transformation.

## 1. Introduction

Low-carbon structural steel is widely used in construction and engineering structures [1]. Some companies have begun to explore the use of low-carbon steel plates to manufacture machine tool beds by welding. However, complex residual stress is generated during the manufacturing process owing to misfits in the shape of parts of both different regions and different phases, or even because of local variations in elastic constants, or thermal and mechanical properties, which are self-equilibrating stresses that remain in the part after manufacturing [2,3]. Annealing is a heat treatment method for releasing stress and improving microstructure when the steel is heated to an approximate Ac1 temperature and then slowly cooled in the furnace to obtain a near-equilibrium structure [4,5].

Researchers have investigated the effects of peak temperature, heating and cooling rates, and holding time on the microstructure and properties of annealing [6,7,8,9]. The yield strength and ultimate tensile strength are inversely correlated with the annealing temperature owing to grain growth, whereas elongation is positively correlated with the annealing temperature owing to the austenite stability caused by carbide dissolution [10,11]. Moreover, dislocation density decreases with increasing annealing temperature [12]. Although the effect of holding time on the microstructure of steel is weaker than the peak temperature, the microstructure of steel will coarsen with time, and properties show random behavior, increasing its variance as aging time increases [13,14,15]. At the same time, the cooling rate also has a great effect on the microstructure evolution. A multi-phase structure composed of ferrite, martensite, retained austenite, and carbides can be obtained by adjusting the cooling rate of low alloy steel [16]. With the increase of cooling rate, the onset temperature of phase transformation of pearlite steel decreases, while the content of pearlite increases and the structure refines (both lamella spacing and cementite lamella thickness are reduced) [17,18]. Rapid heating with high temperatures can not only refine the grains and improve the strength but also affect the distribution and morphology of austenite due to the overlapping of ferrite recrystallization and austenite formation processes [19].

Furthermore, stress also has a significant effect on microstructure evolution during annealing [20,21,22]. Hydrostatic pressure can offset the chemical driving energy to inhibit volume expansion transformation, such as ferrite, pearlite, bainite, and martensite transformation, and even austenite-ferrite reverse transformation [23,24,25]. Uniaxial stress enhances ferrite and pearlite transformation because it can act as an additional driving force [26,27]. The face-centered cubic structure of austenite is transformed into body-centered cubic ferrite through a local rearrangement of atoms, which is an energy-consuming process of coupling a/γ interface migration and element partitioning, where the value depends on the interface mobility [28]. Stress has different effects according to the growth direction of ferrite and changes the activation energy [29,30].

Q235 steel (Steel Brand of China) is a mild steel widely used in construction and engineering structures, and the study of its kinetics of phase transformation under stress is of great significance to the annealing process. Therefore, this work focuses on the effect of both temperatures and uniaxial compressive stress on the kinetics of phase transformation in Q235 steel. A detailed analysis is conducted using a modified JMA kinetics model containing an impingement parameter. Activation energies are also well-discussed and related to the transformation mechanisms.

## 2. Materials and Methods

**Figure 1:**
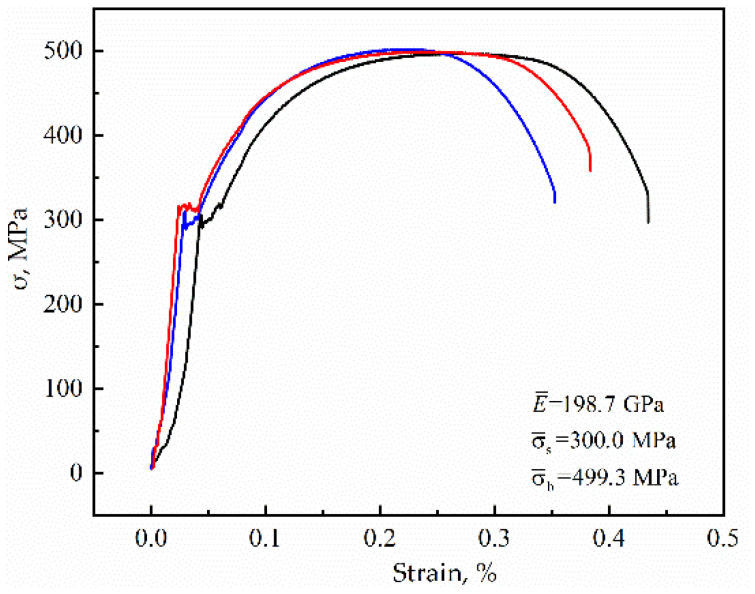
Engineering stress-strain curve.

The material is Q235 steel (Steel Brand of China), and its composition is given in Table 1. The tensile properties of this steel are shown in Figure 1, where the modulus of elasticity is 198.7 GPa, the yield strength is 300 MPa, and the tensile strength is 499.3 MPa. 

The Gleeble mechanical system is a complete, fully integrated hydraulic servo system capable of exerting static force in dilatation, tension, compression, or torsion and can be achieved using transducers, load cells, or non-contact laser extensometry to provide feedback to ensure accurate execution and repeatability of the mechanical test program. To simulate the microstructure evolution under stress using the phase-transformation kinetics model of steel and provide theoretical guidance for determining the technological parameters of annealing is one of the aims. The dilatometric curves of machined cylindrical Q235 steel samples with a diameter of 10 mm and length of 15 mm were measured using a Gleeble-3500 thermal-mechanical simulator at cooling rates of 25 and 50 K/min (to ensure that the microstructure after annealing is pearlite and ferrite). Additionally, when cooled to 1063 K, the stress is maintained until the specimen is cooled to room temperature. The applied stresses were 15 and 30 MPa.

**Figure 2:**
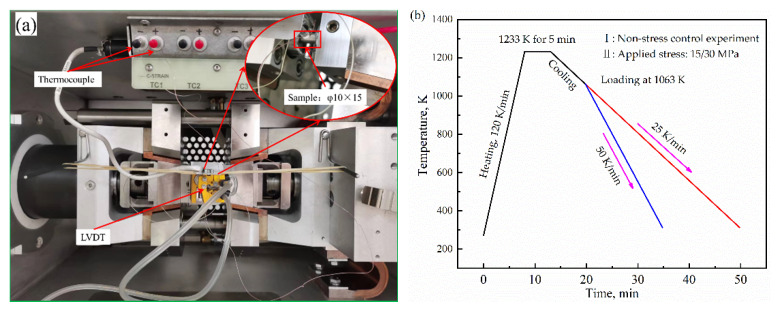
Experimental procedure. (**a**) photos of the experimental process; (**b**) scheme of the proposed thermomechanical cycles.

The schematic of the thermomechanical treatment is shown in Figure 2. The change in the diameter was measured using a linear variable differential transformer (LVDT). The diameter change calibration was also performed for the pure austenite and ferrite phases during the transformations to eliminate the elastic deformation of the specimen after applying compressive stress [30].

After being cut by wire electrical discharge machining, the samples were polished with 400–1500 mesh sandpaper. The microstructure was characterized by field emission scanning electron microscopy (GeminiSEM 500) together with a Nordlys HKL-Oxford EBSD camera. For the EBSD study, the specimens were prepared by electropolishing in a solution of 4% perchloric acid and alcohol at a potential of 20 V for 15 s. EBSD examinations were performed at a potential voltage of 20 kV and a scanning step size of 0.5 µm. The microhardness was measured under a load of 300-gf and a dwell time of 15 s using a Vickers hardness tester (Tukon 2500 Wilson, Norwood, MA, USA). Each hardness value presented is the average of the five indentations.

## 3. Results and Discussion

### 3.1. Microstructure Evolution

**Figure 3:**
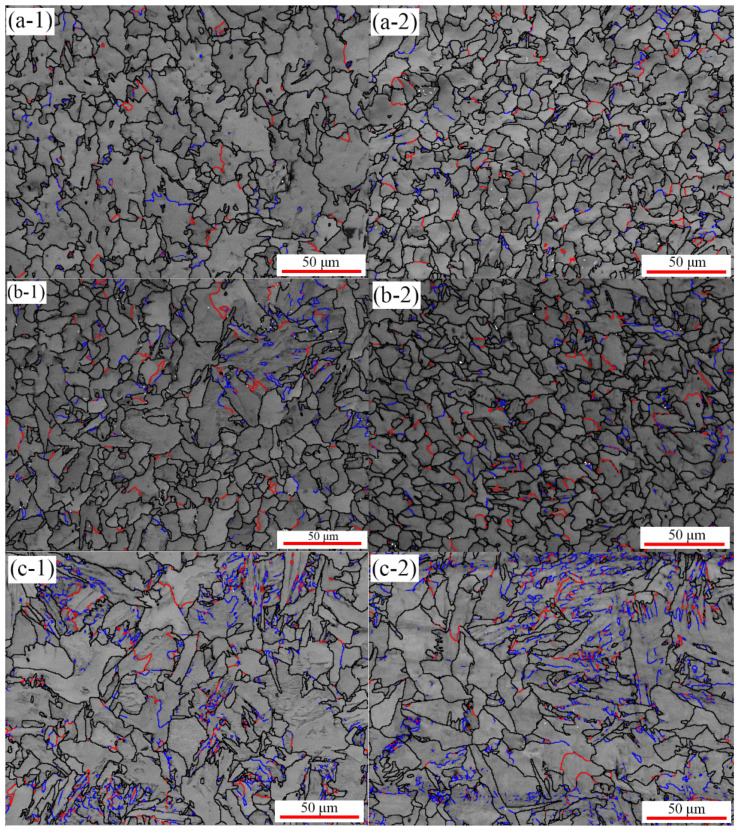
EBSD band contrast maps with grain boundary misorientation distribution of Q235 (blue: BRA < 5°; red: 5° < BRA < 15°, black: 15° < BRA). (**a-1**–**c-1**) are cooled samples at 25 K/min and (**a-2**–**c-2**) are cooled samples at 50 K/min. Red and blue lines indicate the low-angle boundaries, black lines indicate high-angle boundaries, respectively.

**Figure 4:**
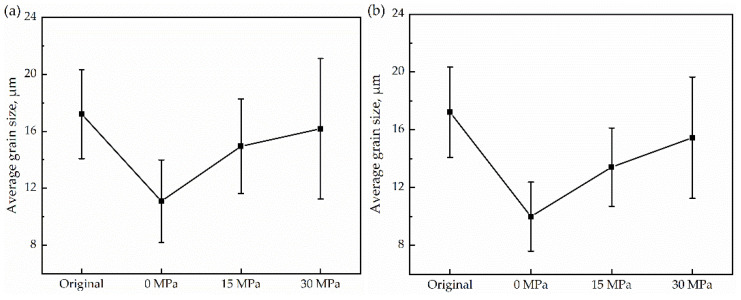
The average grain radius is determined by the line-intercept method. (**a**) 25 K/min; (**b**) 50 K/min.

The microstructure was obtained by applying a scanning electron microscope equipped with a device for electron backscatter diffraction and Channel 5 software for the analysis of the Kikuchi pattern. The EBSD band contrast maps with grain boundary misorientation distributions exhibiting microstructural evolution are shown in Figure 2. Three levels are set according to the angle of the grain boundary misorientation distribution, which are marked as blue for less than 5°, red for 5–15°, and black for marks above 15°. As depicted in Figure 3(a-1,a-2), the microstructure was dominated by equiaxed crystals, whose dimensions were approximately the same in all directions. With increasing uniaxial compressive stress, both grain size variability and low-angle grain boundaries increase, as shown in Figure 3(c-1,c-2) and Figure 4. When the material is under the action of stress, the atoms move away from the equilibrium position to produce a small displacement, and the binding energy also increases with increasing displacement. Under continuous stress, the slip band inside the matrix is activated, dislocations increase, and a large number of small-angle grain boundaries are formed. The appearance of a large number of low-angle boundaries indicated that the specimen underwent local plastic deformation as the stress increased [30]. This is because the sum of the internal stress generated by the transformation and the external compressive stress locally exceeds the yield stress of ferrite, resulting in local non-uniform plastic deformation of the specimen.

The average grain radius is calculated by the line-intercept method. For the samples, Figure 3 were used for the calculations. Four lines (one vertical, one horizontal, and two diagonal) were drawn in each image. The average number of intercepts and the average line length were calculated. Then, the grain size was calculated. As shown in Figure 4, the grain size decreases after thermal cycling compared with the original sample. However, grain size increases with the increase of stress. In addition, the distribution of grains became heterogeneous with increasing stress. During the phase transformation, ferrite nucleates preferentially at the austenite grain boundaries, which limits the formation of the initial ferrite nuclei, and the difference in the number of original grains in the sample is small. In addition, the uniaxial compressive stress provides extra energy for grain growth leading to coarsening. However, the new grains between the corners of the coarsened grains are inhibited from growing, leading to a larger variance of average grain size.

**Figure 5:**
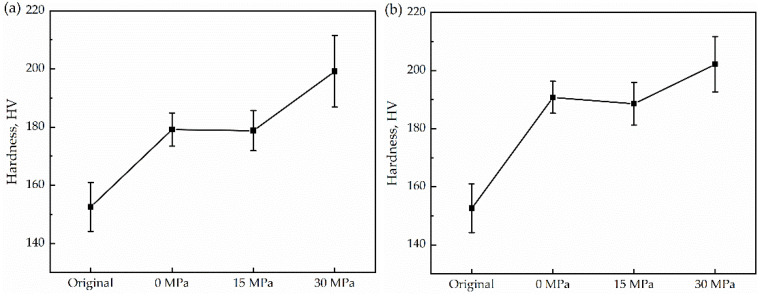
Evolution of Microhardness. (**a**) 25 K/min; (**b**) 50 K/min.

Another general technique for investigating the microstructure is studying the evolution of microhardness, where the performance can be easily calculated based on the hardness values [31]. Figure 5a shows that the microhardness of the original sample was 152.6 HV0.3. At the cooling rate of 25 K/min, the hardness of the sample increases from 179.2 HV0.3 to 199.2 HV0.3, an increase of 11.2%. Figure 5b displayed that at the cooling rate of 50 K/min, the hardness increases from 190.8 HV0.3 to 202.2 HV0.3, an increase of 5.97%. The hardness measured by the tester indicates the difficulty of pressing the indenter into the metal [32,33]. This is the difficulty in the plastic deformation of the metal. Although the grains coarsened as the compressive stress increased, the density of low-angle grain boundaries increased with increasing stress, as shown in Figure 3, which has a positive effect on the hardness value because they are capable of hindering dislocation motion and causing strengthening [34,35]. The low-angle boundaries formed present obstacles to the motion of gliding dislocations, which are, thus, forced to take a bowed configuration. As a consequence, the long-range internal back stresses arise in the grains and forward stresses at the boundaries. It is more difficult for the dislocations to squeeze through low-angle boundaries than through high ones, which leads to hardening. This is the reason why the hardness increases slightly although the grains are coarsened. 

### 3.2. Phase-Transformation Kinetics Using the Modified JMA Model

The volume fraction of ferrite was evaluated using the lever principle according to the dilatometry curves. Suppose that the size change of the sample is isotropic in the phase transformation process, and a small cubic region is taken from the sample. The value is sufficiently small, such that the value of $3d_{0}\Delta d^{2}+\Delta d^{3}$ is close to zero and can be ignored without affecting the calculation. Then, the relationship between the relative volume change $\Delta V/V_{0}$ and the relative length $\Delta d/d_{0}$ can be expressed as [36]:

(1) $$\{\begin{array}{ll} \Delta V & =V-V_{0}={\left(d_{0}+\Delta d\right)}^{3}-d_{0}^{3}\approx 3d_{0}^{2}\Delta d\\ & \frac{\Delta V}{V_{0}}=\frac{3d_{0}^{2}\Delta d}{V_{0}}=\frac{3d_{0}^{2}\Delta d}{d_{0}^{3}}=3\frac{\Delta d}{d_{0}} \end{array}$$

where $d_{0}$ is the initial diameter of the sample, Δd is the diameter change, $V_{0}$ is the initial volume of the sample, and ΔV is the volume change.

**Figure 6:**
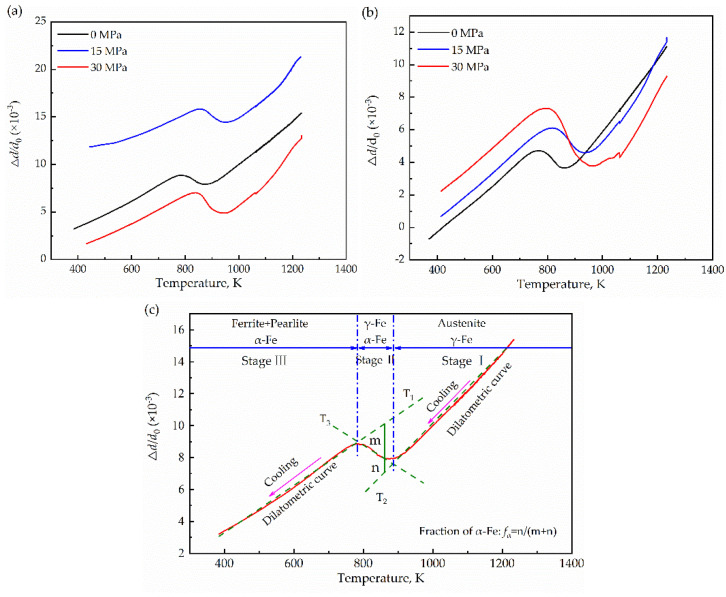
Dilatometric curves of specimens during cooling. (**a**,**b**) dilatometric curves of samples at 25 K/min, 50 K/min, respectively. (**c**) schematic diagram of the dilatometric curve, tangent-intersection method, and the level principle (0 MPa−25 K/min).

According to Equation (1), the volume fraction of ferrite can be evaluated by the change in the length of the sample during the cooling process, as shown in Figure 6c. The dilatometry curves of the samples are divided into three stages. Stage I indicates that the austenite shrinks with decreasing temperature, and stage II represents the transformation from austenite to ferrite during continuous cooling, because the specific volume of ferrite (the body-centered cubic) is greater than austenite (the face-centered cubic), and the volume expands during the formation process. Stage III is the cold shrinkage of the transformation products with cooling.

According to Figure 6c, the volume fraction of ferrite was calculated using the level principle as follows:

(2) $$f_{\alpha }=\frac{n}{m+n}=\frac{f_{1}T-\left(k_{2}T+p_{2}\right)}{\left(k_{1}T+p_{1}\right)-\left(k_{2}T+p_{2}\right)}$$

where *f*_α_ is the volume fraction of ferrite, *k*_1_ and *k*_2_ are the slopes of tangent lines *T*_1_ and *T*_2_, *p*_1_ and *p*_2_ are the intercepts of tangent lines *T*_1_ and T_2_, and *f*_1_ is the dilatometric curve.

**Figure 7:**
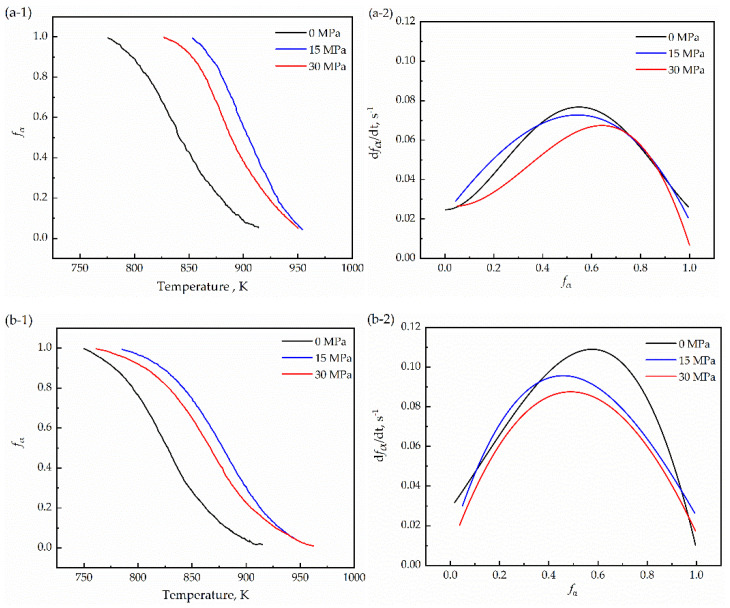
Ferrite volume fraction via lever principle (**a-1**,**b-1**), and ferrite-formation rate (**a-2**,**b-2**) of the sample as a function of temperature under cooling rate of 25 K/min, 50 K/min, respectively.

Figure 7(a-1,b-1) shows the curves of the ferrite transformation fraction with temperature calculated by the lever principle (Equation (2)). The relationship between the volume of ferrite and the transformation rate was obtained by the derivative of the transformation volume fraction to time, and the obtained data were fitted using a quartic polynomial, as shown in Figure 7(a-2,b-2). In the figure, the maximum phase transformation rate of the transformation decreases with an increase in compressive stress. In addition, there is only one peak during the phase transformation, which means that the normal phase transformation occurs under compressive stress. This is in good agreement with the results reported by Liu et al. [37]. It is observed that the higher the stress, the lower the transformation rate. The effect of stress on the transformation kinetics can be interpreted based on the microstructure during thermomechanical treatment. Increasing the stress from 0 to 30 MPa modified the local interface, increased its grain size, and reduced the density of the α/γ boundary.

A general procedure for simulating phase transformation kinetics based on nucleation, growth, and impingement mechanisms has been described in Refs. [38,39]. Johnson–Mehl–Avrami (JMA) nucleation and growth kinetics are some of the most useful models for evaluating the volume fraction of a new phase transformed in the heating or cooling process. The volume of all the growing particles was first calculated, assuming that the grains grew and new nuclei were generated in the material. Owing to the larger degree of under cooling, it can be considered that nucleation of ferrite can take place upon traversing the two-phase region. All the nuclei began to grow without any obvious continuous nucleation. Therefore, all nuclei are present and start growing, and the nucleation rate per unit volume is expressed as:

(3) $$\dot{N}\left(t\right)=N^{*}\delta \left(\frac{T\left(t\right)-T_{0}}{\phi }\right)$$

where $N^{*}$ is the density of nuclei per unit volume and its relationship with the average grain size *d* is $N^{*}=d^{-3}$, d is the diameter of ferrite, ϕ is the cooling rate, and $\delta \left(\frac{T\left(t\right)-T_{0}}{\phi }\right)$ is the Dirac function.

In the JMA model, it is assumed that the grains are randomly generated when the driving force is large or the crystal structure is very close to the matrix. Owing to the inhomogeneity of the grain size and shape, the dispersion of the grown particles is also aperiodic. Thus, the dispersion of growing particles can be considered “pseudo-random”. The modification of relative variables according to different implementation effects can be expressed as [37].

(4) h(fα)={I(fα)=dfαdt=1−fα Randomly nuclei, istropic growthI(fα)=dfαdt=(1−fα2) Non-randomly nucleiI(fα)=dfαdt=(1−fα)2 Anistropic growth

Nucleation is preferred at the grain corners, so the dispersion of growing particles cannot be considered to be truly “random” [40]. Moreover, heterogeneous nucleation is more common because it can effectively reduce the interfacial energy and nucleation barrier. In addition, anisotropic grain growth was observed. For a non-random nucleation distribution, there is a situation between random nucleation and periodic nucleation, so the relationship between *f_α_* and *X_e_* is [37,41,42]:

(5) $$X_{e}=arctanh(f_{\alpha })\;=arctan\left(1-f_{\alpha }^{2}\right)$$

where *X_e_* is the extended volume. The extended volume *(X_e_ = V_e_/V*) is equal to the ratio of the extended volume of the particles to the sample volume (*V*).

The transformation rate is expressed by interface-controlled growth and saturation nucleation:

(6) dfαdt=dfαdxe·dxedt=I(fα)3(gN*)13(xe(fα))23vα

where g is a geometrical factor and g = 4π/3.

So, the interface migration can be evaluated:

(7) vα=dfαdt/I(fα)3(gN*)13(xe(fα))23

**Figure 8:**
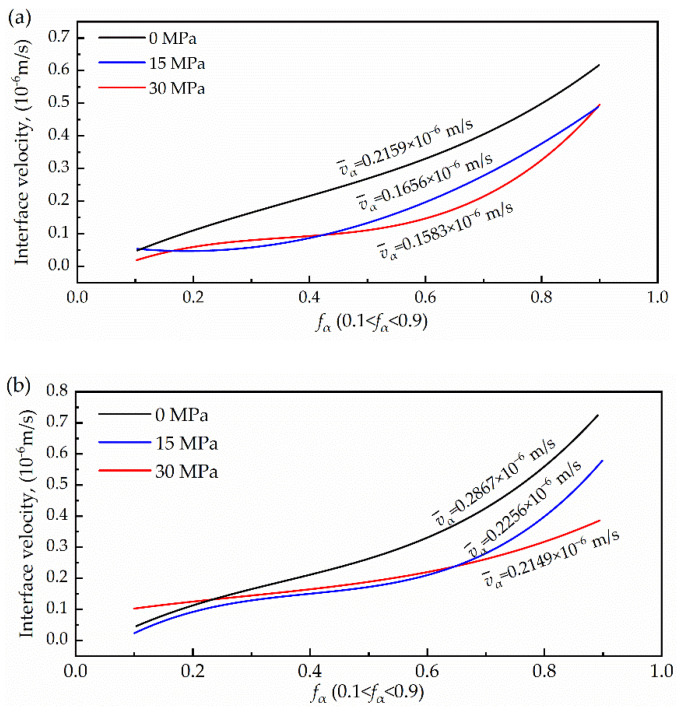
The interface migration velocity as determined for the indicated applied compressive stress. (**a**) 25 K/min; (**b**) 50 K/min.

The results $v_{\alpha }$ for $f_{\alpha }$ < 0.1 and $f_{\alpha }$ > 0.9 are not reliable because at these values of $f_{\alpha }$, the left- and right-hand sides of Equation (7) become vanishingly small and independent of the interface velocity, and because of the accumulation and release of the mismatch energy, the calculation results fluctuate, and a cubic polynomial is used to fit these curves to obtain smooth curves, as shown in Figure 8. The interface migration velocity is determined by the phase structure formed by the generated grain and parent phase. At the initial stage of phase transformation, due to the small grain size of the new phase, the surface energy will play a predominant function, and the crystal nucleus and the parent phase form a coherent interface to reduce the surface energy. With the growth of the new phase grains, the elastic strain energy may exceed the yield limit of the parent phase and produce plastic deformation, which leads to the destruction of the coherent interface. Therefore, the new phase grains may form semi-coherent or non-coherent interfaces with the parent phase during the continuous growth process. Therefore, the growth of the new phase is mainly accomplished by the collective thermal activation transition, which is different from the growth of the new phase crystal nucleus by a single atomic thermal activation transition process. As such, Figure 8 shows that the interface migration velocity increases with the increase of transition.

In the figure, when the stress increases from 0 MPa to 30 MPa, the interfacial migration velocity decreases from 0.2159 to 0.1583 μm/s at 25 K/min, a decrease of 36.4%. When the cooling rate is 10 K/min, the interfacial migration velocity decreases from 0.2867 to 0.2149 μm/s, a 33.4% decrease. The results showed that the interface migration velocity decreased with an increase in the compressive stress. This is because the orientation relationship between the grain boundaries is an important factor affecting the phase-transition rate [34,43,44,45]. The structure of the low-angle grain boundaries is tighter, and interface migration is determined by the diffusion of vacancies between dislocations [46]. However, the structure of the high-angle grain boundary is relatively loose, the orientation difference between the grain boundaries is large, and its movement is controlled by the jumping of atoms at the interface. Moreover, the activation energy of the high-angle grain boundary was significantly lower than that of the low-angle grain boundary, and the migration activity of the phase interface was greater. This is precisely due to the reduction in the high-angle grain boundaries under the action of stress, where the interface migration rate decreases with the increase in stress. It can be concluded that the compressive stress inhibits the interfacial migration velocity, thereby reducing the phase transformation rate.

Comparing Figure 8a,b, it can be observed that when the cooling rate was 50 K/min, the interfacial migration velocity was greater than 25 K/min. High temperature atoms vibrate violently because of higher energy. When the temperature decreases, atoms do not return to the equilibrium position to cause lattice vacancies. As the cooling rate increased, vacancy-dominated defects in the system increased, and these defects were annihilated at the phase interface, resulting in a looser interface structure. As a result, the energy barrier that the parent atoms overcome by thermal activation crossing the interface into the new phase will be reduced. Moreover, with an increase in defects, the average atomic migration distance decreases. Ultimately, the above reasons lead to an increase in interfacial migration velocity.

### 3.3. Driving Force for the Transformation of Austenite to Ferrite

The phase transformation expansion volume can be expressed as:

(8) $$V_{e}=N^{*}Vg{\left(\int_{0}^{t}v_{\alpha }dt\right)}^{3}$$

**Figure 9:**
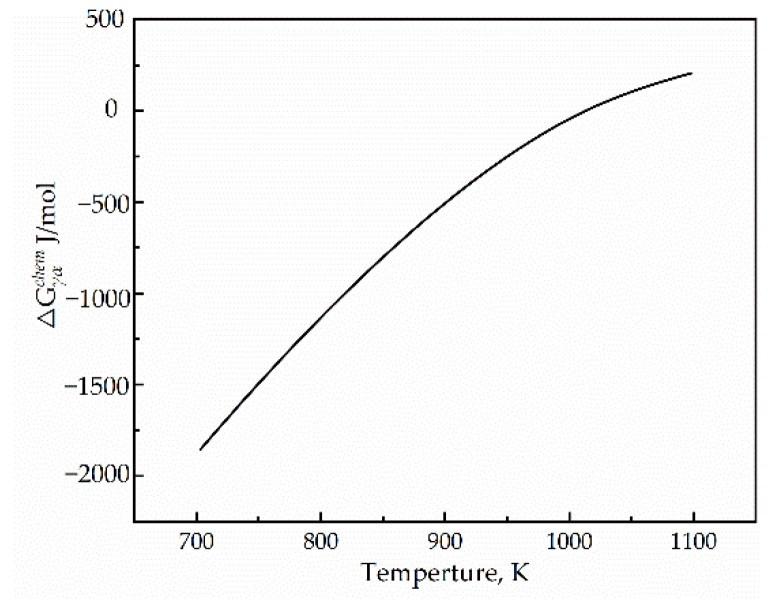
The chemical energy difference calculated by JMatPro.

The interface migration velocity is proportional to the product of the interface mobility (*M*) and driving force:

(9) $$v_{\alpha }=M\left(T\right)\left(-\Delta G_{\alpha \gamma }\left(T,f_{\alpha }\right)\right)$$

when *M(T)* is the interface mobility, it exhibits an Arrhenius-like temperature dependence; the interface driving force ($-\Delta G_{\alpha \gamma }\left(T,f_{\alpha }\right)$) includes the temperature-dependent chemical Gibbs energy difference between ferrite and austenite, and ($\Delta G_{\alpha \gamma }^{chem}\left(T\right)$ was calculated by JMatPro, as shown in Figure 9, which provides the driving force for the phase transition, and the sum energy resulting from misfit strains between the parent and product phases and the γ/α interface energy ($\left[\Delta G_{\alpha \gamma }^{def}\left(f_{\alpha }\right)+\Delta G_{\alpha \gamma }^{int}\left(f_{\alpha }\right)\right]$), which hinders transformation and increases with the fraction of transformation. Therefore, the negative value of the interface driving force can be expressed as:

(10) $$\Delta G_{\alpha \gamma }\left(T,f_{\alpha }\right)=\Delta G_{\alpha \gamma }^{chem}\left(T\right)+\left[\Delta G_{\alpha \gamma }^{def}\left(f_{\alpha }\right)+\Delta G_{\alpha \gamma }^{int}\left(f_{\alpha }\right)\right]$$

The interface migration is calculated by the Arrhenius expression:

(11) $$M=M_{0}\exp\left(-Q/RT\right)$$

where $M_{0}$ is the pre-exponential constant of the JMA equation and *Q* is the activation of the transformation. A large variation in the $M_{0}$ value slightly affects the calculated values of the sum of the $\Delta G_{\alpha \gamma }^{def}\left(f_{\alpha }\right)+\Delta G_{\alpha \gamma }^{int}\left(f_{\alpha }\right)$ [37]. Thus, it is reasonable to adopt the mobility data of pure iron ($M=4900\exp\left(-147000/RT\right)\;\mathrm{mmolJ}{}^{-1}s^{-1}$) to express the γ/α interface migration [47].

**Figure 10:**
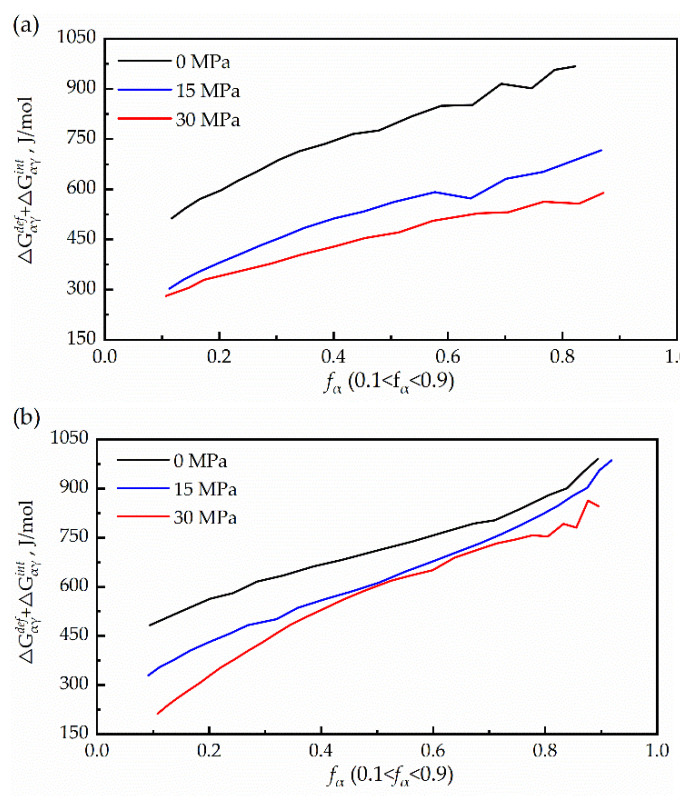
The sum of misfit-accommodation energy and interface energy as a function of transformed fraction. (**a**) 25 K/min; (**b**) 50 K/min.

According to Equations (9) and (10), the sum of the misfit accommodation energy and interface energy versus the transformed fraction ($\Delta G_{\alpha \gamma }^{def}\left(f_{\alpha }\right)+\Delta G_{\alpha \gamma }^{int}\left(f_{\alpha }\right)$ can be calculated, as shown in Figure 10. This is of the same order of magnitude as $\Delta G_{\alpha \gamma }^{chem}\left(T\right)$. Moreover, it decreased significantly with an increase in the applied stress. It can be concluded that compressive stress promotes the phase transition by providing additional energy of the phase transformation. 

## 4. Conclusions

This study examined the transformation and kinetics of the phase transformation of Q235 structural steel using Gleeble 3500 under variable uniaxial compressive stress. The microstructural evolution of Q235 steel with ferrite and pearlite structures during thermomechanical treatment was systematically investigated, and its microhardness was determined. The following conclusions can be drawn from this work.

1. Observing the microstructure, it was found that the grain size after thermal cycling was slightly finer than that of the original sample. Low-angle grain boundaries also increase with increasing stress. Meanwhile, additional energy was supplied to the grain growth, resulting in a decrease in the nucleation number and gradual coarsening. In addition, the microhardness increased with uniaxial compressive stress.
2. The application of stress led to an increase in the grain size and a decrease in the density of the α/γ boundary, resulting in a decrease in the interfacial migration velocity with increasing stress. Therefore, the maximum transformation rate decreases with increasing compressive stress, and the single peak indicates that the transformation mode is a normal austenite-ferrite transformation under stress.
3. Most of the chemical Gibbs free energy is dissipated by the sum of the misfit accommodation energy and interface energy during the γ→α transformation. The driving force of the γ→α transformation was very small compared with the sum energy.

## Figures and Tables

**Table 1:** Chemical composition (in weight %).

Composition	C	Si	Mn	P	S	Cr	Fe
Content	0.15	0.14	0.71	0.03	0.03	0.24	Bal.

## Data Availability

Data available on request from the corresponding authors.
